# Undeca­carbonyl-1κ^3^ *C*,2κ^4^ *C*,3κ^4^ *C*-{tris­[4-(methyl­sulfanyl)­phen­yl]arsine-1κ*As*}-*triangulo*-triruthenium(0)

**DOI:** 10.1107/S1600536810029223

**Published:** 2010-08-04

**Authors:** Omar bin Shawkataly, Imthyaz Ahmed Khan, Siti Syaida Sirat, Chin Sing Yeap, Hoong-Kun Fun

**Affiliations:** a Chemical Sciences Programme, School of Distance Education, Universiti Sains Malaysia, 11800 USM, Penang, Malaysia; b X-ray Crystallography Unit, School of Physics, Universiti Sains Malaysia, 11800 USM, Penang, Malaysia

## Abstract

The crystal structure of the title *triangulo*-triruthenium compound, [Ru_3_(C_21_H_21_AsS_3_)(CO)_11_], confirms that during the synthesis one equatorial carbonyl ligand is substituted by a monodentate arsine ligand, leaving one equatorial and two axial carbonyl substituents on an Ru atom. The other two Ru atoms each carry two equatorial and two axial carbonyl ligands. The three arsine-substituted benzene rings make dihedral angles of 77.94 (13), 86.37 (13) and 73.22 (12)° with each other. Two of the methylsulfanyl groups are disordered over two positions with refined site occupancies of 0.720 (7):0.280 (7) and 0.644 (8):0.356 (8). In the crystal structure, mol­ecules are linked into infinite chains along the *a* axis by weak inter­molecular C—H⋯O hydrogen bonds.

## Related literature

For general background to *triangulo*-triruthenium derivatives, see: Bruce *et al.* (1985 ▶, 1988*a* ▶,*b* ▶). For related structures, see: Shawkataly *et al.* (1998 ▶, 2004 ▶, 2009 ▶, 2010 ▶). For the stability of the temperature controller used in the data collection, see: Cosier & Glazer (1986 ▶). 
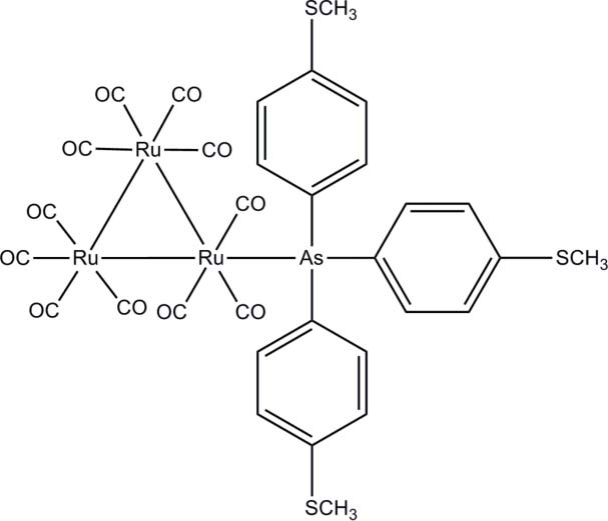


## Experimental

### 

#### Crystal data

[Ru_3_(C_21_H_21_AsS_3_)(CO)_11_]
*M* *_r_* = 1055.80
Monoclinic, 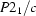

*a* = 14.3855 (2) Å
*b* = 15.1185 (2) Å
*c* = 19.0966 (3) Å
β = 118.221 (1)°
*V* = 3659.57 (9) Å^3^
*Z* = 4
Mo *K*α radiation
μ = 2.35 mm^−1^
*T* = 100 K
0.51 × 0.16 × 0.12 mm

#### Data collection

Bruker SMART APEXII CCD area-detector diffractometer
Absorption correction: multi-scan (*SADABS*; Bruker, 2009 ▶) *T*_min_ = 0.378, *T*_max_ = 0.768
71424 measured reflections
16330 independent reflections
11811 reflections with *I* > 2σ(*I*)
*R*_int_ = 0.034

#### Refinement

*R*[*F* ^2^ > 2σ(*F* ^2^)] = 0.036
*wR*(*F* ^2^) = 0.095
*S* = 1.02
16330 reflections
494 parameters
H-atom parameters constrained
Δρ_max_ = 1.99 e Å^−3^
Δρ_min_ = −0.85 e Å^−3^

### 

Data collection: *APEX2* (Bruker, 2009 ▶); cell refinement: *SAINT* (Bruker, 2009 ▶); data reduction: *SAINT*; program(s) used to solve structure: *SHELXTL* (Sheldrick, 2008 ▶); program(s) used to refine structure: *SHELXTL*; molecular graphics: *SHELXTL*; software used to prepare material for publication: *SHELXTL* and *PLATON* (Spek, 2009 ▶).

## Supplementary Material

Crystal structure: contains datablocks global, I. DOI: 10.1107/S1600536810029223/lh5089sup1.cif

Structure factors: contains datablocks I. DOI: 10.1107/S1600536810029223/lh5089Isup2.hkl

Additional supplementary materials:  crystallographic information; 3D view; checkCIF report

## Figures and Tables

**Table 1:** Hydrogen-bond geometry (Å, °)

*D*—H⋯*A*	*D*—H	H⋯*A*	*D*⋯*A*	*D*—H⋯*A*
C8—H8*A*⋯O10^i^	0.93	2.46	3.317 (3)	153
C20—H20*A*⋯O6^ii^	0.96	2.59	3.153 (4)	118

Symmetry codes: (i) 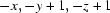; (ii) 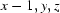.

**Fig. 1.**
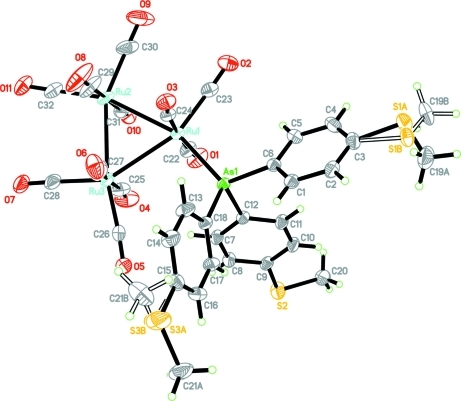
The molecular structure of the title compound with 50% probability ellipsoids for non-H atoms. All disorder component are shown.

**Fig. 2.**
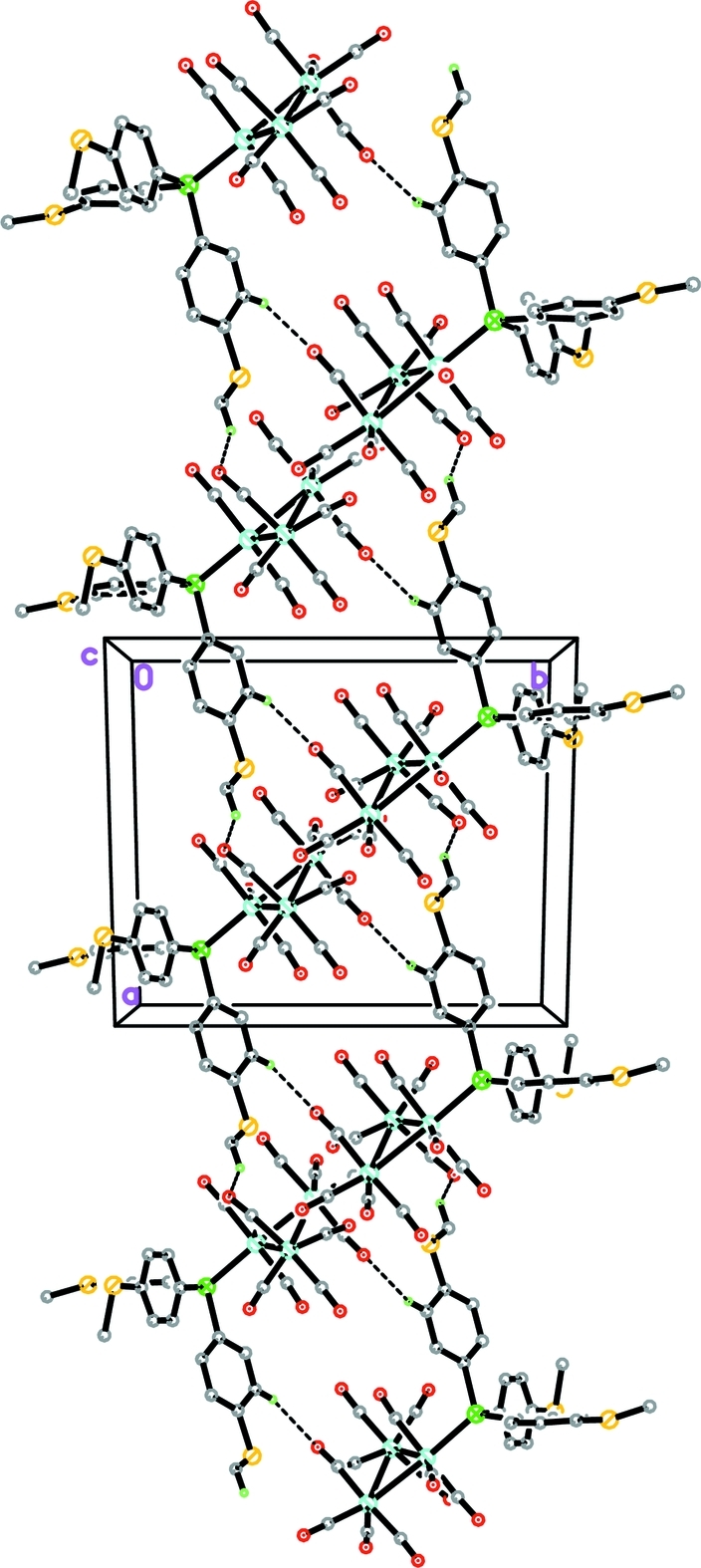
The crystal packing of the title compound, viewed down the c axis, showing the molecules linked into a 1-D chain along the a axis. The minor disorder component and hydrogen atoms not involved in the hydrogen-bonding (dashed lines) have been omitted for clarity.

## supplementary crystallographic information

### Comment

*Triangulo*-triruthenium clusters are known for their interesting structural variations and related catalytic activity. A large number of substituted derivatives, Ru_3_(CO)_12-n_*L*_n_ (*L*= group 15 ligand) have been reported (Bruce *et al.*, 1988*a*, *b*; Bruce *et al.*, 1985). As part of our study on the substitution of transition metal-carbonyl clusters with mixed-ligand complexes (Shawkataly *et al.*, 1998, 2004, 2009, 2010), herein we report the synthesis and structure of title compound.

In the title molecule (Fig. 1), a monodentate arsine ligand has replaced a single carbonyl ligand of the Ru_3_ triangle. The monodentate arsine ligand is bonded equatorially to atom Ru1 of the *triangulo*-triruthenium. Atoms Ru2 and Ru3 each carry two equatorial and two axial terminal carbonyl ligands. The three arsine-substituted benzene rings make dihedral angles (C1–C6/C7–C12, C1–C6/C13–C18 and C7–C12/C13–C18) of 77.94 (13), 86.37 (13) and 73.22 (12)° with each other respectively.

In the crystal structure, the molecules are linked into dimers by intermolecular C8—H8A···O10^i^ hydrogen bonds (Fig. 2, Table 1). These dimers are further linked into infinite one-dimensional chains along the *a* axis by weak intermolecular C20—H20A···O6^ii^ hydrogen bonds (Fig. 2, Table 1).

### Experimental

The reactions were conducted under an atmosphere of high purity nitrogen using standard Schlenk techniques and tetrahydrofuran (THF) dried over sodium metal. Tris(4-(methylsulfanyl)phenyl)arsine was prepared by the reaction of AsCl_3_ with 4-SCH_3_C_6_H_4_MgBr in THF. Equimolar quantity of Ru_3_(CO)_12_ and tris(4-(methylsulfanyl)phenyl)arsine were stirred in THF (25 ml) under nitrogen. About 0.2 ml of diphenylketyl radical anion initiator was introduced into the reaction mixture under a current of nitrogen. The reaction mixture turned intense red. After 10 minutes of stirring the solvent was removed under vacuum. The reaction mixture was separated by TLC (acetone:hexane, 10:90). Three bands appeared. The major band (red) *R*_f_=0.78 was separated and characterized. Single crystals of title compound were crystallized from CH_2_Cl_2_—CH_3_OH.

### Refinement

All hydrogen atoms were positioned geometrically and refined using a riding model with C—H = 0.93 or 0.96 Å and *U*_iso_(H) = 1.2 or 1.5*U*_eq_(C). The rotating group model was applied to the methyl groups. Two of the methylsulfanyl groups are disordered over two positions with site occupancies of 0.720 (7)/0.280 (7) and 0.644 (8)/0.356 (8).

### Crystal data

**Table:**

[Ru_3_(C_21_H_21_AsS_3_)(CO)_11_]	*F*(000) = 2056
*M**_r_* = 1055.80	*D*_x_ = 1.916 Mg m^−^^3^
Monoclinic, *P*2_1_/*c*	Mo *K*α radiation, λ = 0.71073 Å
Hall symbol: -P 2ybc	Cell parameters from 9931 reflections
*a* = 14.3855 (2) Å	θ = 2.1–35.2°
*b* = 15.1185 (2) Å	µ = 2.35 mm^−^^1^
*c* = 19.0966 (3) Å	*T* = 100 K
β = 118.221 (1)°	Block, yellow
*V* = 3659.57 (9) Å^3^	0.51 × 0.16 × 0.12 mm
*Z* = 4	

### Data collection

**Table:**

Bruker SMART APEXII CCD area-detector diffractometer	16330 independent reflections
Radiation source: fine-focus sealed tube	11811 reflections with *I* > 2σ(*I*)
graphite	*R*_int_ = 0.034
φ and ω scans	θ_max_ = 35.3°, θ_min_ = 1.8°
Absorption correction: multi-scan (*SADABS*; Bruker, 2009)	*h* = −19→23
*T*_min_ = 0.378, *T*_max_ = 0.768	*k* = −24→24
71424 measured reflections	*l* = −30→30

### Refinement

**Table:**

Refinement on *F*^2^	Primary atom site location: structure-invariant direct methods
Least-squares matrix: full	Secondary atom site location: difference Fourier map
*R*[*F*^2^ > 2σ(*F*^2^)] = 0.036	Hydrogen site location: inferred from neighbouring sites
*wR*(*F*^2^) = 0.095	H-atom parameters constrained
*S* = 1.02	*w* = 1/[σ^2^(*F*_o_^2^) + (0.0418*P*)^2^ + 4.1199*P*] where *P* = (*F*_o_^2^ + 2*F*_c_^2^)/3
16330 reflections	(Δ/σ)_max_ = 0.002
494 parameters	Δρ_max_ = 1.99 e Å^−^^3^
0 restraints	Δρ_min_ = −0.85 e Å^−^^3^

### Special details

**Table:**

Experimental. The crystal was placed in the cold stream of an Oxford Cryosystems Cobra open-flow nitrogen cryostat (Cosier & Glazer, 1986) operating at 100.0 (1) K.
Geometry. All e.s.d.'s (except the e.s.d. in the dihedral angle between two l.s. planes) are estimated using the full covariance matrix. The cell e.s.d.'s are taken into account individually in the estimation of e.s.d.'s in distances, angles and torsion angles; correlations between e.s.d.'s in cell parameters are only used when they are defined by crystal symmetry. An approximate (isotropic) treatment of cell e.s.d.'s is used for estimating e.s.d.'s involving l.s. planes.
Refinement. Refinement of *F*^2^ against ALL reflections. The weighted *R*-factor *wR* and goodness of fit *S* are based on *F*^2^, conventional *R*-factors *R* are based on *F*, with *F* set to zero for negative *F*^2^. The threshold expression of *F*^2^ > σ(*F*^2^) is used only for calculating *R*-factors(gt) *etc*. and is not relevant to the choice of reflections for refinement. *R*-factors based on *F*^2^ are statistically about twice as large as those based on *F*, and *R*- factors based on ALL data will be even larger.

### Fractional atomic coordinates and isotropic or equivalent isotropic displacement parameters (Å^2^)

**Table:**

	*x*	*y*	*z*	*U*_iso_*/*U*_eq_	Occ. (<1)
Ru1	0.303224 (14)	0.708502 (12)	0.663222 (11)	0.02112 (4)	
Ru2	0.443037 (15)	0.566710 (13)	0.683937 (13)	0.02879 (5)	
Ru3	0.303059 (15)	0.625745 (13)	0.526436 (11)	0.02490 (5)	
As1	0.179968 (17)	0.830239 (15)	0.597008 (13)	0.01887 (5)	
S1A	0.15141 (11)	1.13790 (18)	0.82192 (13)	0.0415 (5)	0.720 (7)
S1B	0.1413 (3)	1.1668 (3)	0.7944 (3)	0.0287 (9)	0.280 (7)
S2	−0.31571 (5)	0.71815 (5)	0.42081 (5)	0.03608 (15)	
S3A	0.2279 (3)	1.0400 (3)	0.3187 (2)	0.0598 (9)	0.644 (8)
S3B	0.2214 (5)	1.0245 (3)	0.3115 (4)	0.0276 (8)	0.356 (8)
O1	0.11132 (17)	0.59537 (15)	0.63507 (15)	0.0452 (5)	
O2	0.34832 (18)	0.72081 (15)	0.83481 (12)	0.0399 (5)	
O3	0.48841 (14)	0.83567 (13)	0.70489 (12)	0.0323 (4)	
O4	0.12039 (19)	0.50166 (17)	0.50092 (14)	0.0514 (6)	
O5	0.14841 (19)	0.71935 (15)	0.37430 (12)	0.0408 (5)	
O6	0.4620 (2)	0.77043 (17)	0.54294 (15)	0.0471 (6)	
O7	0.38667 (18)	0.48066 (16)	0.45883 (14)	0.0463 (6)	
O8	0.62067 (18)	0.68968 (17)	0.6949 (2)	0.0680 (9)	
O9	0.5349 (3)	0.56039 (19)	0.86415 (15)	0.0796 (11)	
O10	0.26753 (17)	0.44371 (13)	0.67228 (12)	0.0341 (4)	
O11	0.55046 (17)	0.40839 (15)	0.65245 (15)	0.0443 (5)	
C1	0.18435 (18)	1.01529 (16)	0.64286 (14)	0.0241 (4)	
H1A	0.1898	1.0287	0.5974	0.029*	
C2	0.17614 (19)	1.08362 (17)	0.68915 (16)	0.0294 (5)	
H2A	0.1768	1.1422	0.6746	0.035*	
C3	0.1671 (2)	1.06389 (19)	0.75696 (16)	0.0316 (5)	
C4	0.1700 (2)	0.9761 (2)	0.77931 (15)	0.0328 (6)	
H4A	0.1656	0.9624	0.8251	0.039*	
C5	0.1794 (2)	0.90880 (17)	0.73399 (14)	0.0262 (4)	
H5A	0.1824	0.8504	0.7501	0.031*	
C6	0.18441 (17)	0.92752 (15)	0.66463 (13)	0.0217 (4)	
C7	−0.00677 (18)	0.73374 (16)	0.48932 (14)	0.0244 (4)	
H7A	0.0398	0.7043	0.4763	0.029*	
C8	−0.11261 (18)	0.71044 (16)	0.45143 (15)	0.0258 (4)	
H8A	−0.1366	0.6663	0.4129	0.031*	
C9	−0.18313 (17)	0.75325 (16)	0.47118 (14)	0.0244 (4)	
C10	−0.14614 (19)	0.82007 (17)	0.52802 (15)	0.0270 (5)	
H10A	−0.1925	0.8492	0.5414	0.032*	
C11	−0.03987 (19)	0.84348 (16)	0.56490 (15)	0.0253 (4)	
H11A	−0.0160	0.8886	0.6024	0.030*	
C12	0.03098 (17)	0.80031 (15)	0.54641 (13)	0.0213 (4)	
C13	0.28856 (19)	0.91950 (19)	0.52287 (16)	0.0306 (5)	
H13A	0.3482	0.9088	0.5712	0.037*	
C14	0.2990 (2)	0.9631 (2)	0.46368 (17)	0.0348 (6)	
H14A	0.3651	0.9822	0.4724	0.042*	
C15	0.2102 (2)	0.97864 (18)	0.39047 (16)	0.0307 (5)	
C16	0.1120 (2)	0.95100 (18)	0.37907 (15)	0.0310 (5)	
H16A	0.0523	0.9615	0.3307	0.037*	
C17	0.10259 (19)	0.90789 (17)	0.43939 (14)	0.0266 (5)	
H17A	0.0364	0.8899	0.4313	0.032*	
C18	0.19080 (17)	0.89119 (14)	0.51187 (13)	0.0204 (4)	
C19A	0.1225 (4)	1.2377 (3)	0.7657 (3)	0.0517 (14)	0.720 (7)
H19A	0.0999	1.2821	0.7903	0.078*	0.720 (7)
H19B	0.0674	1.2270	0.7127	0.078*	0.720 (7)
H19C	0.1846	1.2578	0.7638	0.078*	0.720 (7)
C19B	0.0958 (10)	1.1186 (8)	0.8589 (7)	0.041 (3)	0.280 (7)
H19D	0.0653	1.1638	0.8770	0.062*	0.280 (7)
H19E	0.1542	1.0920	0.9038	0.062*	0.280 (7)
H19F	0.0437	1.0743	0.8304	0.062*	0.280 (7)
C20	−0.3707 (2)	0.7605 (2)	0.48061 (17)	0.0380 (6)	
H20A	−0.4421	0.7401	0.4599	0.057*	
H20B	−0.3699	0.8240	0.4797	0.057*	
H20C	−0.3298	0.7401	0.5343	0.057*	
C21A	0.0914 (6)	1.0606 (6)	0.2413 (4)	0.088 (3)	0.644 (8)
H21A	0.0922	1.0914	0.1976	0.132*	0.644 (8)
H21B	0.0557	1.0959	0.2630	0.132*	0.644 (8)
H21C	0.0553	1.0052	0.2230	0.132*	0.644 (8)
C21B	0.3528 (8)	1.0789 (7)	0.3593 (5)	0.046 (3)	0.356 (8)
H21D	0.4069	1.0348	0.3828	0.069*	0.356 (8)
H21E	0.3568	1.1188	0.3997	0.069*	0.356 (8)
H21F	0.3627	1.1113	0.3200	0.069*	0.356 (8)
C22	0.1840 (2)	0.63313 (17)	0.64345 (17)	0.0304 (5)	
C23	0.3311 (2)	0.71844 (17)	0.77005 (16)	0.0292 (5)	
C24	0.42108 (18)	0.78543 (16)	0.68645 (13)	0.0241 (4)	
C25	0.1903 (2)	0.54647 (19)	0.51609 (16)	0.0354 (6)	
C26	0.2055 (2)	0.68672 (18)	0.43243 (16)	0.0298 (5)	
C27	0.4067 (2)	0.7162 (2)	0.54203 (17)	0.0337 (6)	
C28	0.3586 (2)	0.5355 (2)	0.48489 (17)	0.0358 (6)	
C29	0.5519 (2)	0.6469 (2)	0.6887 (2)	0.0482 (9)	
C30	0.5025 (3)	0.5640 (2)	0.79728 (19)	0.0496 (9)	
C31	0.3279 (2)	0.49241 (17)	0.67318 (14)	0.0279 (5)	
C32	0.5110 (2)	0.46800 (19)	0.66342 (17)	0.0349 (6)	

### Atomic displacement parameters (Å^2^)

**Table:**

	*U*^11^	*U*^22^	*U*^33^	*U*^12^	*U*^13^	*U*^23^
Ru1	0.01770 (7)	0.01787 (8)	0.02283 (8)	−0.00003 (6)	0.00550 (6)	0.00147 (6)
Ru2	0.01928 (8)	0.02086 (9)	0.03296 (10)	0.00343 (6)	0.00144 (7)	0.00179 (7)
Ru3	0.02032 (8)	0.02407 (9)	0.02616 (9)	0.00328 (6)	0.00759 (7)	−0.00128 (7)
As1	0.01585 (9)	0.01732 (10)	0.02025 (10)	−0.00062 (7)	0.00591 (7)	−0.00037 (7)
S1A	0.0398 (6)	0.0459 (10)	0.0332 (8)	0.0048 (6)	0.0126 (5)	−0.0149 (7)
S1B	0.0354 (14)	0.0205 (14)	0.0340 (18)	−0.0023 (10)	0.0194 (13)	−0.0070 (12)
S2	0.0185 (2)	0.0465 (4)	0.0422 (4)	−0.0062 (2)	0.0136 (2)	−0.0141 (3)
S3A	0.0476 (15)	0.096 (2)	0.0475 (16)	0.0226 (16)	0.0324 (13)	0.0400 (15)
S3B	0.0366 (15)	0.0180 (10)	0.0270 (13)	−0.0077 (9)	0.0141 (10)	−0.0006 (9)
O1	0.0322 (10)	0.0328 (11)	0.0653 (15)	−0.0072 (9)	0.0187 (10)	0.0065 (10)
O2	0.0441 (12)	0.0440 (12)	0.0280 (9)	−0.0035 (9)	0.0140 (8)	0.0056 (8)
O3	0.0236 (8)	0.0315 (9)	0.0343 (9)	−0.0055 (7)	0.0076 (7)	−0.0005 (7)
O4	0.0418 (12)	0.0471 (13)	0.0463 (13)	−0.0171 (10)	0.0054 (10)	0.0021 (10)
O5	0.0484 (12)	0.0421 (12)	0.0313 (10)	0.0182 (10)	0.0184 (9)	0.0060 (8)
O6	0.0493 (13)	0.0502 (14)	0.0566 (14)	−0.0135 (11)	0.0372 (12)	−0.0157 (11)
O7	0.0352 (11)	0.0450 (12)	0.0453 (12)	0.0132 (9)	0.0082 (9)	−0.0149 (10)
O8	0.0220 (10)	0.0402 (13)	0.126 (3)	0.0043 (9)	0.0222 (13)	0.0160 (15)
O9	0.102 (2)	0.0504 (16)	0.0331 (12)	0.0215 (16)	−0.0115 (14)	−0.0067 (11)
O10	0.0381 (10)	0.0284 (9)	0.0358 (10)	−0.0038 (8)	0.0175 (8)	−0.0041 (8)
O11	0.0326 (10)	0.0340 (11)	0.0604 (14)	0.0103 (9)	0.0170 (10)	0.0042 (10)
C1	0.0207 (9)	0.0228 (10)	0.0247 (10)	−0.0012 (8)	0.0075 (8)	−0.0025 (8)
C2	0.0207 (10)	0.0232 (11)	0.0366 (13)	−0.0001 (8)	0.0071 (9)	−0.0081 (9)
C3	0.0212 (10)	0.0389 (14)	0.0320 (12)	−0.0036 (9)	0.0103 (9)	−0.0154 (10)
C4	0.0303 (12)	0.0445 (15)	0.0236 (11)	−0.0045 (11)	0.0127 (9)	−0.0080 (10)
C5	0.0275 (11)	0.0287 (11)	0.0221 (10)	−0.0028 (9)	0.0115 (8)	−0.0014 (8)
C6	0.0182 (9)	0.0215 (10)	0.0219 (9)	−0.0016 (7)	0.0065 (7)	−0.0024 (7)
C7	0.0187 (9)	0.0244 (10)	0.0293 (11)	−0.0014 (8)	0.0107 (8)	−0.0061 (8)
C8	0.0199 (9)	0.0250 (11)	0.0308 (11)	−0.0034 (8)	0.0107 (8)	−0.0073 (9)
C9	0.0159 (8)	0.0260 (11)	0.0286 (11)	0.0002 (8)	0.0085 (8)	−0.0010 (9)
C10	0.0194 (9)	0.0286 (11)	0.0320 (12)	0.0034 (8)	0.0112 (9)	−0.0041 (9)
C11	0.0232 (10)	0.0204 (10)	0.0305 (11)	0.0006 (8)	0.0113 (9)	−0.0041 (8)
C12	0.0183 (8)	0.0194 (9)	0.0239 (9)	−0.0011 (7)	0.0082 (7)	−0.0006 (8)
C13	0.0186 (9)	0.0385 (14)	0.0305 (12)	0.0009 (9)	0.0082 (9)	0.0102 (10)
C14	0.0268 (11)	0.0443 (15)	0.0373 (13)	0.0052 (11)	0.0184 (10)	0.0125 (12)
C15	0.0375 (13)	0.0320 (13)	0.0284 (11)	0.0056 (10)	0.0205 (10)	0.0041 (10)
C16	0.0304 (12)	0.0334 (13)	0.0217 (10)	−0.0002 (10)	0.0061 (9)	0.0022 (9)
C17	0.0204 (9)	0.0283 (11)	0.0245 (10)	−0.0008 (8)	0.0053 (8)	0.0005 (9)
C18	0.0175 (8)	0.0195 (9)	0.0214 (9)	0.0002 (7)	0.0070 (7)	−0.0002 (7)
C19A	0.049 (3)	0.038 (2)	0.060 (3)	0.011 (2)	0.019 (2)	−0.014 (2)
C19B	0.056 (7)	0.039 (6)	0.045 (6)	−0.009 (5)	0.038 (6)	−0.018 (5)
C20	0.0217 (11)	0.0568 (19)	0.0373 (14)	0.0036 (11)	0.0154 (10)	0.0004 (13)
C21A	0.076 (5)	0.154 (8)	0.056 (4)	0.075 (5)	0.049 (4)	0.070 (5)
C21B	0.048 (5)	0.056 (6)	0.038 (4)	−0.030 (4)	0.024 (4)	−0.015 (4)
C22	0.0268 (11)	0.0225 (11)	0.0387 (13)	−0.0009 (9)	0.0128 (10)	0.0026 (10)
C23	0.0262 (11)	0.0232 (11)	0.0324 (12)	−0.0014 (9)	0.0089 (9)	0.0039 (9)
C24	0.0196 (9)	0.0244 (10)	0.0227 (10)	0.0005 (8)	0.0055 (8)	0.0012 (8)
C25	0.0307 (12)	0.0340 (13)	0.0295 (12)	−0.0010 (11)	0.0045 (10)	0.0033 (10)
C26	0.0310 (12)	0.0309 (12)	0.0308 (12)	0.0063 (10)	0.0173 (10)	−0.0019 (10)
C27	0.0325 (12)	0.0368 (14)	0.0372 (13)	−0.0020 (11)	0.0208 (11)	−0.0092 (11)
C28	0.0241 (11)	0.0380 (14)	0.0339 (13)	0.0042 (10)	0.0044 (10)	−0.0052 (11)
C29	0.0221 (12)	0.0287 (13)	0.077 (2)	0.0076 (10)	0.0096 (13)	0.0065 (14)
C30	0.0492 (18)	0.0264 (13)	0.0391 (15)	0.0092 (12)	−0.0072 (13)	−0.0034 (11)
C31	0.0300 (11)	0.0232 (11)	0.0242 (10)	0.0038 (9)	0.0077 (9)	−0.0015 (8)
C32	0.0240 (11)	0.0290 (12)	0.0409 (14)	0.0032 (10)	0.0065 (10)	0.0055 (11)

### Geometric parameters (Å, °)

**Table:**

Ru1—C23	1.890 (3)	C2—C3	1.394 (4)
Ru1—C24	1.927 (2)	C2—H2A	0.9300
Ru1—C22	1.941 (3)	C3—C4	1.389 (4)
Ru1—As1	2.4515 (3)	C4—C5	1.383 (4)
Ru1—Ru2	2.8363 (3)	C4—H4A	0.9300
Ru1—Ru3	2.8953 (3)	C5—C6	1.389 (3)
Ru2—C30	1.915 (3)	C5—H5A	0.9300
Ru2—C32	1.923 (3)	C7—C8	1.387 (3)
Ru2—C31	1.930 (3)	C7—C12	1.392 (3)
Ru2—C29	1.949 (3)	C7—H7A	0.9300
Ru2—Ru3	2.8583 (3)	C8—C9	1.397 (3)
Ru3—C26	1.913 (3)	C8—H8A	0.9300
Ru3—C28	1.931 (3)	C9—C10	1.391 (3)
Ru3—C27	1.939 (3)	C10—C11	1.393 (3)
Ru3—C25	1.950 (3)	C10—H10A	0.9300
As1—C18	1.938 (2)	C11—C12	1.389 (3)
As1—C6	1.938 (2)	C11—H11A	0.9300
As1—C12	1.943 (2)	C13—C14	1.377 (4)
S1A—C3	1.762 (3)	C13—C18	1.388 (3)
S1A—C19A	1.783 (6)	C13—H13A	0.9300
S1B—C19B	1.799 (12)	C14—C15	1.397 (4)
S1B—C3	1.823 (4)	C14—H14A	0.9300
S2—C9	1.763 (2)	C15—C16	1.388 (4)
S2—C20	1.787 (3)	C16—C17	1.385 (4)
S3A—C15	1.771 (5)	C16—H16A	0.9300
S3A—C21A	1.842 (8)	C17—C18	1.389 (3)
S3B—C15	1.736 (7)	C17—H17A	0.9300
S3B—C21B	1.858 (10)	C19A—H19A	0.9600
O1—C22	1.135 (3)	C19A—H19B	0.9600
O2—C23	1.141 (3)	C19A—H19C	0.9600
O3—C24	1.147 (3)	C19B—H19D	0.9600
O4—C25	1.131 (4)	C19B—H19E	0.9600
O5—C26	1.135 (3)	C19B—H19F	0.9600
O6—C27	1.137 (4)	C20—H20A	0.9600
O7—C28	1.135 (4)	C20—H20B	0.9600
O8—C29	1.140 (4)	C20—H20C	0.9600
O9—C30	1.135 (4)	C21A—H21A	0.9600
O10—C31	1.133 (3)	C21A—H21B	0.9600
O11—C32	1.136 (4)	C21A—H21C	0.9600
C1—C6	1.390 (3)	C21B—H21D	0.9600
C1—C2	1.400 (3)	C21B—H21E	0.9600
C1—H1A	0.9300	C21B—H21F	0.9600
			
C23—Ru1—C24	89.36 (11)	C6—C5—H5A	119.6
C23—Ru1—C22	88.50 (12)	C5—C6—C1	119.1 (2)
C24—Ru1—C22	177.65 (12)	C5—C6—As1	118.71 (18)
C23—Ru1—As1	103.28 (8)	C1—C6—As1	121.99 (18)
C24—Ru1—As1	90.50 (7)	C8—C7—C12	121.2 (2)
C22—Ru1—As1	89.05 (8)	C8—C7—H7A	119.4
C23—Ru1—Ru2	97.75 (8)	C12—C7—H7A	119.4
C24—Ru1—Ru2	86.29 (7)	C7—C8—C9	120.0 (2)
C22—Ru1—Ru2	94.95 (8)	C7—C8—H8A	120.0
As1—Ru1—Ru2	158.685 (11)	C9—C8—H8A	120.0
C23—Ru1—Ru3	156.56 (8)	C10—C9—C8	119.2 (2)
C24—Ru1—Ru3	95.38 (7)	C10—C9—S2	124.06 (19)
C22—Ru1—Ru3	86.97 (9)	C8—C9—S2	116.71 (18)
As1—Ru1—Ru3	99.628 (9)	C9—C10—C11	120.2 (2)
Ru2—Ru1—Ru3	59.816 (7)	C9—C10—H10A	119.9
C30—Ru2—C32	102.42 (13)	C11—C10—H10A	119.9
C30—Ru2—C31	90.59 (14)	C12—C11—C10	120.9 (2)
C32—Ru2—C31	91.14 (11)	C12—C11—H11A	119.5
C30—Ru2—C29	92.33 (17)	C10—C11—H11A	119.5
C32—Ru2—C29	90.99 (13)	C11—C12—C7	118.5 (2)
C31—Ru2—C29	175.93 (12)	C11—C12—As1	122.26 (17)
C30—Ru2—Ru1	94.72 (10)	C7—C12—As1	119.22 (17)
C32—Ru2—Ru1	162.43 (8)	C14—C13—C18	121.3 (2)
C31—Ru2—Ru1	84.77 (7)	C14—C13—H13A	119.3
C29—Ru2—Ru1	92.17 (9)	C18—C13—H13A	119.3
C30—Ru2—Ru3	155.78 (10)	C13—C14—C15	119.9 (3)
C32—Ru2—Ru3	101.59 (9)	C13—C14—H14A	120.0
C31—Ru2—Ru3	85.85 (7)	C15—C14—H14A	120.0
C29—Ru2—Ru3	90.32 (11)	C16—C15—C14	119.2 (2)
Ru1—Ru2—Ru3	61.117 (7)	C16—C15—S3B	119.3 (3)
C26—Ru3—C28	102.56 (11)	C14—C15—S3B	121.4 (3)
C26—Ru3—C27	88.52 (13)	C16—C15—S3A	123.0 (2)
C28—Ru3—C27	97.09 (12)	C14—C15—S3A	117.7 (3)
C26—Ru3—C25	89.71 (12)	C17—C16—C15	120.3 (2)
C28—Ru3—C25	90.59 (13)	C17—C16—H16A	119.9
C27—Ru3—C25	172.31 (12)	C15—C16—H16A	119.9
C26—Ru3—Ru2	167.72 (8)	C16—C17—C18	120.8 (2)
C28—Ru3—Ru2	89.39 (8)	C16—C17—H17A	119.6
C27—Ru3—Ru2	87.17 (9)	C18—C17—H17A	119.6
C25—Ru3—Ru2	93.04 (8)	C13—C18—C17	118.5 (2)
C26—Ru3—Ru1	109.00 (8)	C13—C18—As1	119.83 (17)
C28—Ru3—Ru1	148.44 (8)	C17—C18—As1	121.63 (18)
C27—Ru3—Ru1	83.35 (8)	S1B—C19B—H19D	109.5
C25—Ru3—Ru1	90.17 (9)	S1B—C19B—H19E	109.5
Ru2—Ru3—Ru1	59.066 (7)	H19D—C19B—H19E	109.5
C18—As1—C6	101.99 (10)	S1B—C19B—H19F	109.5
C18—As1—C12	101.60 (9)	H19D—C19B—H19F	109.5
C6—As1—C12	100.90 (9)	H19E—C19B—H19F	109.5
C18—As1—Ru1	117.66 (7)	S2—C20—H20A	109.5
C6—As1—Ru1	115.98 (7)	S2—C20—H20B	109.5
C12—As1—Ru1	116.14 (7)	H20A—C20—H20B	109.5
C3—S1A—C19A	100.6 (2)	S2—C20—H20C	109.5
C19B—S1B—C3	97.5 (4)	H20A—C20—H20C	109.5
C9—S2—C20	103.61 (13)	H20B—C20—H20C	109.5
C15—S3A—C21A	102.7 (3)	S3B—C21B—H21D	109.5
C15—S3B—C21B	103.9 (4)	S3B—C21B—H21E	109.5
C6—C1—C2	120.2 (2)	H21D—C21B—H21E	109.5
C6—C1—H1A	119.9	S3B—C21B—H21F	109.5
C2—C1—H1A	119.9	H21D—C21B—H21F	109.5
C3—C2—C1	120.1 (2)	H21E—C21B—H21F	109.5
C3—C2—H2A	119.9	O1—C22—Ru1	173.4 (3)
C1—C2—H2A	119.9	O2—C23—Ru1	177.2 (2)
C4—C3—C2	119.2 (2)	O3—C24—Ru1	173.4 (2)
C4—C3—S1A	112.7 (2)	O4—C25—Ru3	172.1 (3)
C2—C3—S1A	128.1 (2)	O5—C26—Ru3	176.2 (2)
C4—C3—S1B	132.7 (3)	O6—C27—Ru3	172.9 (3)
C2—C3—S1B	107.9 (3)	O7—C28—Ru3	176.7 (3)
C5—C4—C3	120.6 (3)	O8—C29—Ru2	175.2 (3)
C5—C4—H4A	119.7	O9—C30—Ru2	177.5 (4)
C3—C4—H4A	119.7	O10—C31—Ru2	173.4 (2)
C4—C5—C6	120.8 (2)	O11—C32—Ru2	178.3 (3)
C4—C5—H5A	119.6		
			
C23—Ru1—Ru2—C30	−5.57 (14)	C23—Ru1—As1—C6	23.31 (11)
C24—Ru1—Ru2—C30	83.28 (14)	C24—Ru1—As1—C6	−66.15 (11)
C22—Ru1—Ru2—C30	−94.73 (15)	C22—Ru1—As1—C6	111.54 (12)
As1—Ru1—Ru2—C30	165.11 (12)	Ru2—Ru1—As1—C6	−147.20 (8)
Ru3—Ru1—Ru2—C30	−178.30 (12)	Ru3—Ru1—As1—C6	−161.70 (8)
C23—Ru1—Ru2—C32	161.7 (3)	C23—Ru1—As1—C12	−94.95 (11)
C24—Ru1—Ru2—C32	−109.4 (3)	C24—Ru1—As1—C12	175.59 (11)
C22—Ru1—Ru2—C32	72.6 (3)	C22—Ru1—As1—C12	−6.72 (12)
As1—Ru1—Ru2—C32	−27.6 (3)	Ru2—Ru1—As1—C12	94.54 (8)
Ru3—Ru1—Ru2—C32	−11.0 (3)	Ru3—Ru1—As1—C12	80.04 (8)
C23—Ru1—Ru2—C31	84.59 (11)	C6—C1—C2—C3	−0.6 (3)
C24—Ru1—Ru2—C31	173.44 (10)	C1—C2—C3—C4	2.2 (4)
C22—Ru1—Ru2—C31	−4.56 (11)	C1—C2—C3—S1A	−177.68 (19)
As1—Ru1—Ru2—C31	−104.73 (8)	C1—C2—C3—S1B	−172.8 (2)
Ru3—Ru1—Ru2—C31	−88.14 (7)	C19A—S1A—C3—C4	−168.0 (3)
C23—Ru1—Ru2—C29	−98.09 (15)	C19A—S1A—C3—C2	11.9 (3)
C24—Ru1—Ru2—C29	−9.24 (14)	C19A—S1A—C3—S1B	−1.3 (4)
C22—Ru1—Ru2—C29	172.75 (15)	C19B—S1B—C3—C4	−10.2 (6)
As1—Ru1—Ru2—C29	72.58 (13)	C19B—S1B—C3—C2	164.0 (5)
Ru3—Ru1—Ru2—C29	89.17 (12)	C19B—S1B—C3—S1A	−26.9 (5)
C23—Ru1—Ru2—Ru3	172.73 (8)	C2—C3—C4—C5	−1.4 (4)
C24—Ru1—Ru2—Ru3	−98.42 (7)	S1A—C3—C4—C5	178.5 (2)
C22—Ru1—Ru2—Ru3	83.58 (9)	S1B—C3—C4—C5	172.2 (3)
As1—Ru1—Ru2—Ru3	−16.59 (3)	C3—C4—C5—C6	−1.0 (4)
C30—Ru2—Ru3—C26	18.5 (5)	C4—C5—C6—C1	2.7 (4)
C32—Ru2—Ru3—C26	−169.0 (4)	C4—C5—C6—As1	−172.47 (19)
C31—Ru2—Ru3—C26	100.6 (4)	C2—C1—C6—C5	−1.9 (3)
C29—Ru2—Ru3—C26	−78.0 (4)	C2—C1—C6—As1	173.11 (17)
Ru1—Ru2—Ru3—C26	14.3 (4)	C18—As1—C6—C5	−179.40 (18)
C30—Ru2—Ru3—C28	−174.8 (3)	C12—As1—C6—C5	76.12 (19)
C32—Ru2—Ru3—C28	−2.30 (13)	Ru1—As1—C6—C5	−50.2 (2)
C31—Ru2—Ru3—C28	−92.62 (12)	C18—As1—C6—C1	5.6 (2)
C29—Ru2—Ru3—C28	88.77 (14)	C12—As1—C6—C1	−98.90 (19)
Ru1—Ru2—Ru3—C28	−178.93 (9)	Ru1—As1—C6—C1	134.74 (17)
C30—Ru2—Ru3—C27	88.1 (3)	C12—C7—C8—C9	−0.9 (4)
C32—Ru2—Ru3—C27	−99.43 (12)	C7—C8—C9—C10	1.1 (4)
C31—Ru2—Ru3—C27	170.25 (11)	C7—C8—C9—S2	−179.2 (2)
C29—Ru2—Ru3—C27	−8.36 (13)	C20—S2—C9—C10	−17.3 (3)
Ru1—Ru2—Ru3—C27	83.93 (8)	C20—S2—C9—C8	162.9 (2)
C30—Ru2—Ru3—C25	−84.2 (3)	C8—C9—C10—C11	−0.3 (4)
C32—Ru2—Ru3—C25	88.26 (13)	S2—C9—C10—C11	180.0 (2)
C31—Ru2—Ru3—C25	−2.06 (12)	C9—C10—C11—C12	−0.7 (4)
C29—Ru2—Ru3—C25	179.33 (14)	C10—C11—C12—C7	0.8 (4)
Ru1—Ru2—Ru3—C25	−88.37 (9)	C10—C11—C12—As1	−179.52 (19)
C30—Ru2—Ru3—Ru1	4.1 (3)	C8—C7—C12—C11	−0.1 (4)
C32—Ru2—Ru3—Ru1	176.63 (9)	C8—C7—C12—As1	−179.7 (2)
C31—Ru2—Ru3—Ru1	86.31 (8)	C18—As1—C12—C11	−107.0 (2)
C29—Ru2—Ru3—Ru1	−92.30 (11)	C6—As1—C12—C11	−2.2 (2)
C23—Ru1—Ru3—C26	164.8 (2)	Ru1—As1—C12—C11	124.06 (19)
C24—Ru1—Ru3—C26	−94.27 (11)	C18—As1—C12—C7	72.6 (2)
C22—Ru1—Ru3—C26	85.67 (12)	C6—As1—C12—C7	177.44 (19)
As1—Ru1—Ru3—C26	−2.85 (9)	Ru1—As1—C12—C7	−56.3 (2)
Ru2—Ru1—Ru3—C26	−176.81 (9)	C18—C13—C14—C15	−0.8 (5)
C23—Ru1—Ru3—C28	−16.3 (3)	C13—C14—C15—C16	1.1 (4)
C24—Ru1—Ru3—C28	84.57 (19)	C13—C14—C15—S3B	−174.3 (3)
C22—Ru1—Ru3—C28	−95.5 (2)	C13—C14—C15—S3A	176.8 (3)
As1—Ru1—Ru3—C28	175.99 (18)	C21B—S3B—C15—C16	164.7 (4)
Ru2—Ru1—Ru3—C28	2.04 (18)	C21B—S3B—C15—C14	−20.0 (5)
C23—Ru1—Ru3—C27	−109.1 (2)	C21B—S3B—C15—S3A	47 (2)
C24—Ru1—Ru3—C27	−8.15 (11)	C21A—S3A—C15—C16	4.9 (5)
C22—Ru1—Ru3—C27	171.79 (12)	C21A—S3A—C15—C14	−170.6 (4)
As1—Ru1—Ru3—C27	83.27 (9)	C21A—S3A—C15—S3B	72 (2)
Ru2—Ru1—Ru3—C27	−90.69 (9)	C14—C15—C16—C17	−0.6 (4)
C23—Ru1—Ru3—C25	75.1 (2)	S3B—C15—C16—C17	174.9 (3)
C24—Ru1—Ru3—C25	175.98 (10)	S3A—C15—C16—C17	−176.0 (3)
C22—Ru1—Ru3—C25	−4.08 (11)	C15—C16—C17—C18	−0.3 (4)
As1—Ru1—Ru3—C25	−92.60 (8)	C14—C13—C18—C17	−0.1 (4)
Ru2—Ru1—Ru3—C25	93.44 (8)	C14—C13—C18—As1	−179.9 (2)
C23—Ru1—Ru3—Ru2	−18.4 (2)	C16—C17—C18—C13	0.7 (4)
C24—Ru1—Ru3—Ru2	82.54 (7)	C16—C17—C18—As1	−179.5 (2)
C22—Ru1—Ru3—Ru2	−97.52 (8)	C6—As1—C18—C13	79.8 (2)
As1—Ru1—Ru3—Ru2	173.957 (10)	C12—As1—C18—C13	−176.2 (2)
C23—Ru1—As1—C18	144.39 (11)	Ru1—As1—C18—C13	−48.3 (2)
C24—Ru1—As1—C18	54.93 (10)	C6—As1—C18—C17	−99.9 (2)
C22—Ru1—As1—C18	−127.38 (11)	C12—As1—C18—C17	4.0 (2)
Ru2—Ru1—As1—C18	−26.12 (8)	Ru1—As1—C18—C17	131.97 (18)
Ru3—Ru1—As1—C18	−40.62 (7)		

### Hydrogen-bond geometry (Å, °)

**Table:**

*D*—H···*A*	*D*—H	H···*A*	*D*···*A*	*D*—H···*A*
C8—H8A···O10^i^	0.93	2.46	3.317 (3)	153.
C20—H20A···O6^ii^	0.96	2.59	3.153 (4)	118.

Symmetry codes: (i) −*x*, −*y*+1, −*z*+1; (ii) *x*−1, *y*, *z*.
